# BioShell-Threading: versatile Monte Carlo package for protein 3D threading

**DOI:** 10.1186/1471-2105-15-22

**Published:** 2014-01-20

**Authors:** Pawel Gniewek, Andrzej Kolinski, Andrzej Kloczkowski, Dominik Gront

**Affiliations:** 1 Laboratory of Theory of Biopolymers, Faculty of Chemistry, University of Warsaw, Pasteura 1, 02-093 Warsaw, Poland; 2 Battelle Center for Mathematical Medicine, The Research Institute at Nationwide Children’s Hospital, 700 Childrens Drive, Columbus, OH 43205, USA; 3 Department of Pediatrics, The Ohio State University

## Abstract

**Background:**

The comparative modeling approach to protein structure prediction inherently relies on a template structure. Before building a model such a template protein has to be found and aligned with the query sequence. Any error made on this stage may dramatically affects the quality of result. There is a need, therefore, to develop accurate and sensitive alignment protocols.

**Results:**

BioShell threading software is a versatile tool for aligning protein structures, protein sequences or sequence profiles and query sequences to a template structures. The software is also capable of sub-optimal alignment generation. It can be executed as an application from the UNIX command line, or as a set of Java classes called from a script or a Java application. The implemented Monte Carlo search engine greatly facilitates the development and benchmarking of new alignment scoring schemes even when the functions exhibit non-deterministic polynomial-time complexity.

**Conclusions:**

Numerical experiments indicate that the new threading application offers template detection abilities and provides much better alignments than other methods. The package along with documentation and examples is available at: http://bioshell.pl/threading3d.

## Background

Protein structure prediction has become one of the key tasks in computational biology of the post-genomic era. Due to the growing size of structural databases, the most important and widely used method is homology modeling. This methodology relies on the existence of structures of homologous protein(s) in databases. The major parts of this procedure are *i) recognition of homology* between two proteins and *ii) correct alignment* for the pair of two proteins for which homology was recognized. Here we focus on the latter, still challenging problem. Accurate alignment is essential for many state-of-the-art 3D protein structure prediction algorithms [1-4]. The development of novel threading algorithms however is hindered by *i)* lack of a general consensus on scoring schemes and *ii)*plethora of different variants of the same scoring function described in literature but not available as a ready-to-use software.

Our contribution presents a versatile tool for the fast and extensive aligning of two proteins with each other. The alignment can be based on *i)*the two sequences, *ii)* one sequence and one structure or *iii)*on the two structures. The first case, corresponding to pairvise sequence alignment is trivial and can be solved by dynamic programming. However, the other two (corresponding to 3D threading and structure alignment, respectively) are NP-hard problems [5]. Our novel object-oriented application, incorporated within the BioShell package [6,7], is an integrated framework to heuristically tackle these protein-to-protein alignment problems. The application is written in Java language which facilitates its easy use on various systems and architectures. The advantages and novelties of the software over the existing and downloadable ones [8-12] are: 

*i) it employs Monte Carlo (MC) to sample the alignment space* so an approximate solution to NP-hard 3D threading problem can be found,

*ii) each scoring term type is a separate object* that can be easily switched on/off and fully customized with user-provided data, e.g. in a single run, several secondary structure similarity scores may be used, each of them based on a different secondary structure prediction,

iii) new potentials and scoring schemes can be easily implemented by users,

*iv) as a result, the user obtains the best scoring solution and a number of suboptimal alignments, ranked by their score*; the alignments can be outputted in the Modeller [1] format file and easily used to build final model structures.

*v) it can be used as a structure alignment software*, also capable of producing suboptimal structure alignments.

*vi) it can read in and score any arbitrary alignment* provided by the user. This can be very helpful in the manual refinement of alignments or for threading force field development.

The project website provides extensive documentation of the library (API) and numerous examples which show how to run the executable threading application and how to interact with the software library.

## Implementation

The source code was divided into four main blocks: *i)* encoding alignment as system coordinates, *ii)* moves (alignment modification), *iii)* scoring and *iv)* gathering results. Each of these components forms a separate sub-package in the source code tree: jbcl.simulations.threading, jbcl.simulations.threading.movers, jbcl.simulations.threading.ff and jbcl.simulations.threading.observers, respectively. These routines are supported by other generic BioShell components such as Monte Carlo sampling and I/O operations. For user’s convenience we provide also a stand-alone application. To run calculations, the user specifies: *i)* input data, *ii)* modification scheme and the of MC sampling and *iii)* scoring function (force field).

### Alignment representation

**Figure 1:**
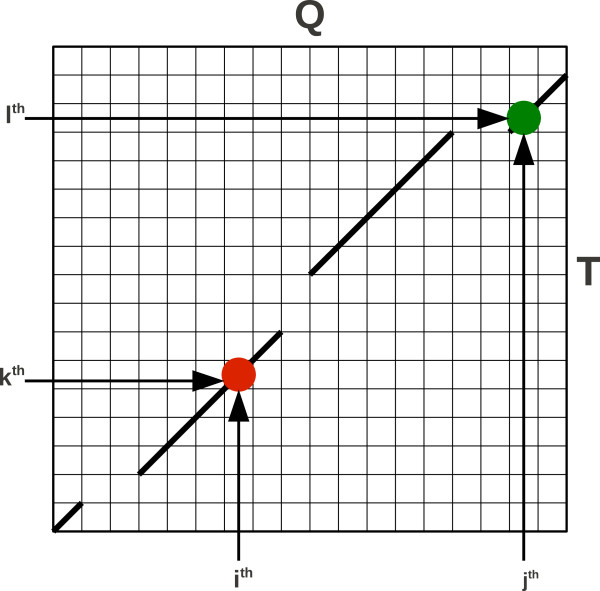
**Graphical representation of an alignment in BioShell-threading.** Portrayed alignment (drawn on an alignment matrix between a query and a template) consists of 4 aligned blocks. Red dot is an alignment of i^*th*^ query’s residue to k^*th*^ template’s residue. Blue dot is an alignment of j^*th*^ query’s residue to k^*th*^ template’s residue.

Protein-to-protein alignment between query (**Q**) and template (**T**) proteins is defined as a list of blocks (see Figure 1 for an example). Every block represents a gapless alignment stretch between a query and a template sequence [13]. An i^th^ block is of the length *L*_*i*_, where the fragment starts at the I^th^ position in the query sequence and at the **J**^th^ position in the template sequence and ends in *I*+*L*_*i*_ and *J*+*L*_*i*_ positions in the query and the template sequences respectively. In the course of code optimization we introduced two restrictions into the program. The first one states that any single block cannot be shorter than the MIN_BLOCK LENGTH which by default is set to 4. Making this value smaller (which can be easily done with a command line option) results in considerably higher computational cost but occasionally might lead to better alignments. The second rule states that an alignment must consist of at least the MIN_NUMBER_OF_BLOCKS, by default equal to 4. This second rule is only a technical trick that gives all movers (see below) a chance to be executed successfully and thus make the sampling process more effective. Since we do not require two neighboring blocks to be separated by a gap, it is always possible to represent a long alignment block as concatenation of several shorter ones. For instance, a perfect alignment of a 100 amino acid sequence with itself may be defined as 25 blocks of 4 residues each, by 4 blocks of 25 residues, or by many other combinations of blocks which are not separated by gaps. All of them however lead to the same alignment and have the same score. Thus the second rule does not limit the sampled conformational space. Moreover, both restricting parameters may be changed by the user from the command line.

### Alignment sampling

**Figure 2:**
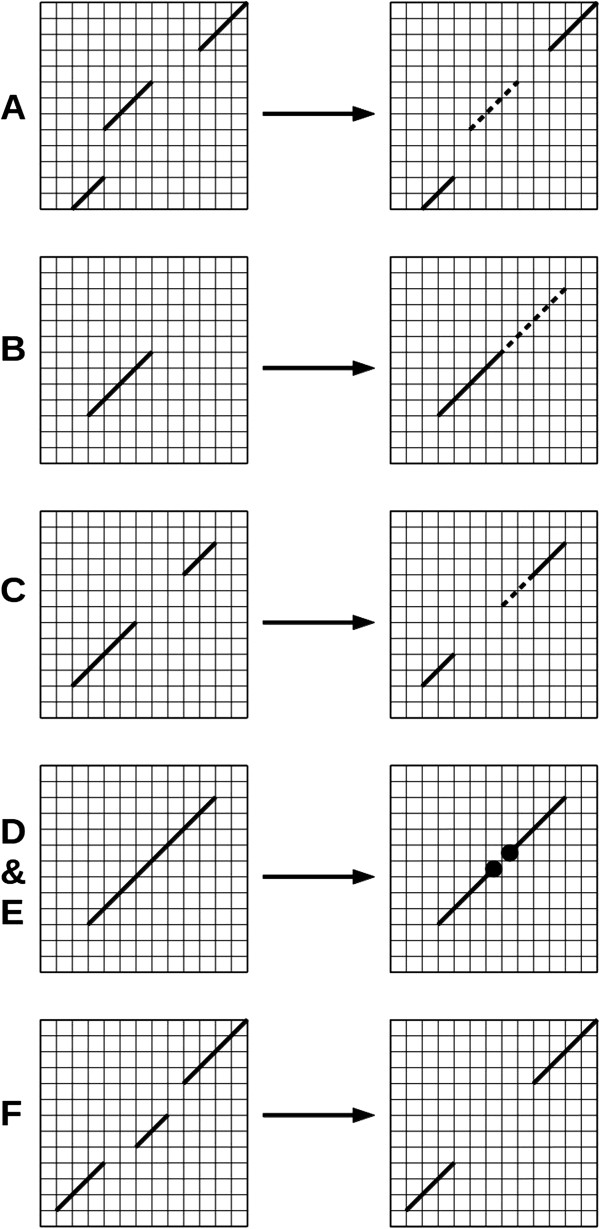
**Different types of moves in BioShell-Threading software.A)** Block Shift **B)** Shrink/Expand **C)** Jump the part of the Block **D&E)** Split/Merge Blocks **F)** Annihilating of Block. For every simulation in this contribution, probability of performing a move by mover was with ratio: 100 : 100 : 100 : 10 : 10 : 1 for A : B : C : D : E : F, respectively. The dashed lines represent moved (panel **A**) and resized (panels **B** and **C**) alignment fragments.

**Figure 3:**
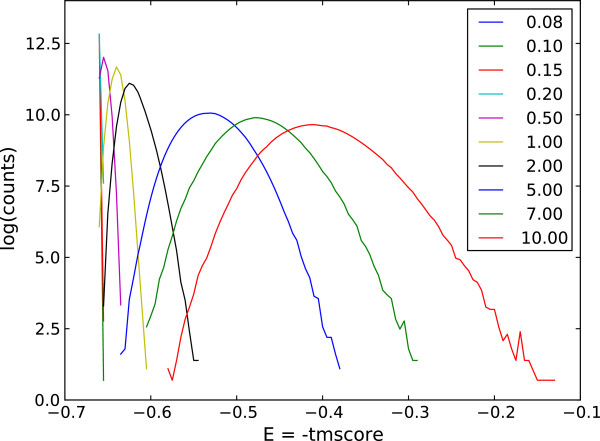
**Energy distributions overlap for 10 replicas in 10 different temperatures.** Counts for every replica are depicted as a logarithm. The temperatures vary from 0.08 to 10 dimensionless units. A significant overlap between replicas suggest that the number and temperatures distribution are chosen correctly.

A list of blocks defines a point in the conformational space of all possible sequence alignments between two proteins. Sampling of this space is performed by a set of movers i.e. objects that attempt to modify an alignment. The following seven types of movers have been implemented so far: Shift a block, Shrink/Expand a block, Merge two blocks, Split one block into two new blocks, Jump part of the one block to a neighbor block, Create a new block and Annihilate a block. The move types have been schematically depicted in the Figure 2. The background grid represents a classic Dynamic Programming (DP) matrix; solid and dashed lines denote an alignment before and after a move, respectively. **BlockShift** shifts a block horizontally and/or vertically on the DP matrix with a uniform distribution within the allowed space (blocks cannot overlap). **BlockShrink & BlockExpand** can shrink or expand a block on either end within the allowed space. A block cannot shrink to a length shorter than the MIN_BLOCK_LENGTH. The length N of shrinking/expanding is generated with 1/2^*N*^ distribution. **BlockPartJump** performs a jump of a part of a block to a neighboring block. The length of the jumping part was generated with uniform distribution which does not violate the MIN_BLOCK_LENGTH. **BlockSplit** can split a block into two parts with conservation of the MIN_BLOCK_LENGTH for both of the two parts. The split is performed with a uniform distribution. **BlocksMerge** can merge two neighboring blocks when possible. Merging continues as long as the MIN_NUMBER_OF_BLOCKS is fulfilled. **BlockAnnihilate** can annihilate one of the alignment blocks if the MIN_NUMBER_OF_BLOCKS is not violated. Finally, **BlockCreate** creates a new block. The user can define how often each of the move types is attempted. Once a mover has been executed and an alignment modified, the new conformation is accepted (or rejected) according to the Metropolis criteria [14]. The sampling process is governed either by Simulated Annealing (SA) [15] or by Replica Exchange Monte Carlo (REMC) [16]. The latter offers a very effective way to sample the conformational space of all possible alignments. As an example (see Figure 3), we show energy distributions obtained by a REMC search comprising ten replicas running at 10 distinct temperatures: 0.08, 0.1, 0.15, 0.2, 0.5, 1.0, 2.0, 5.0, 7.0, 10.0. The distributions exhibit large overlap with neighboring replicas which facilitates random walk in the temperature space and results in highly enhanced sampling. In this particular example 2pcyA query chain was modelled on 2azaA template with (-TMScore) as the energy function. However, we found this set of temperatures to be very universal and working very efficiently for various scoring schemes and different protein lengths. Therefore REMC with these temperature settings (if not stated otherwise) was used for all the numerical experiments described in this contribution.

### Alignment scoring

Each particular alignment is assessed by a scoring function (force field). This function is defined as weighted combination of distinct terms. The scoring terms implemented in the package can be divided into six categories: *(i) structure based* scores, such as TM-score [17] or cRMSD, *(ii) contact potentials* such as Miyazawa-Jerningan [18,19], *(iii) environmental potentials*, e.g. solvent accessibility score *(iv) sequence profile similarity measures*, *(v) secondary structure similarity measures* and *(vi) gap penalty functions.* The full list of available scoring methods is provided in Table 1. Group *(i)* of scores require the query structure to be provided and can be used either for benchmarking purposes (when the query structure is actually known) or for a structure-to-structure Monte Carlo (MC) alignment. Group *(ii)* scores transform a template contact map onto a query sequence based on a current alignment. Finally, scores from groups *(iii)*, *(iv)*, and *(v)* use various kinds of profiles: sequence profiles, predicted propensity for a certain secondary structure type, predicted solvent exposure level etc.

**Table 1:** Potentials implemented within BioShell-threading

**Score type**	**Description**	
AlignemntEnergy	Base class for all alignment scores	
ByAtomEnergy	Score depends solely on a single position in a target and the aligned position in a template	
StructureBasedScore	Knows about target and template atomic coordinates	
BigMatrixEnergy	Pairwise per-position energy may be pre-calculated and stored in a 2D array	
ContactBasedEnergy	Energy that depends on a template contact map	
PairwiseContactEnergy	Contact based energy that is pairwise-decomposable, but can’t be precalculated (otherwise it would result in BigMatrixEnergy score)	
**Score name**	**Derived from**	**References**
DaliScore	StructureBasedScore	[20]
RMSDAlignScore	StructureBasedScore	[21]
TMAlignScore	StructureBasedScore	[17]
GoLikeScore	ContactBased Potential	[22,23]
TwoBodyContact	ContactBasedPotential	[18,19,24-27]
P2PScore	ContactBasedPotential	[28]
StrcGapPenalty	ContactBasedPotential	[29]
AffineGap	AlignmentEnergy	[30]
AsaScore	BigMatrixEnergy	[30]
EnvScore	BigMatrixEnergy	[31]
GravyScore	BigMatrixEnergy	[32]
ProbabilisticSecondaryScore	BigMatrixEnergy	[33]
ProfileScore	BigMatrixEnergy	[34,35]
SubstitutionScore	BigMatrixEnergy	[36]

**Figure 4:**
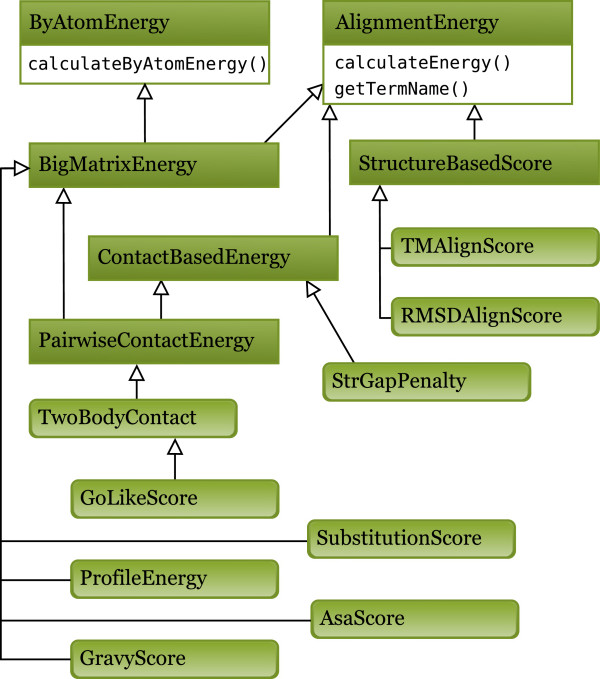
**Energy types inheritance diagram.** All interfaces and the most important scoring types are shown in the form of UML diagram with arrows denoting class inheritance.

An inheritance diagram depicting basic relationships between the classes is shown in the Figure 4, in which each rectangle denotes an abstract interface and a box with rounded corners - an implemented score type. All the score types are derived from AlignmentEnergy. Some of them also inherit from ByAtomEnergy which means that the score value may be decomposed into a sum over all aligned residue pairs. SubstitutionScore based on a substitution matrix such as the BLOSSUM [36] or PAM [37] matrix is obviously a ByAtomEnergy example, while RMSDAlignScore cannot be implemented in this manner. ByAtomEnergy ability is very important for the program as it enables the calculate energy change without the evaluating whole alignment. One a mover has altered an alignment, it returns a list of alignment columns (i.e. the query residue indexes) that have been affected. Then the program evaluates the energy difference at these positions only. Further, some of the ByAtomEnergy scores can be pre-calculated for every query-template residue position once the program is started and stored in a 2D array. Such scores implement a BigMatrixEnergy interface and SubstitutionScore is an example of it. An interesting case is StrGapPenalty derived from ContactBasedEnergy. Indeed, the penalty for a gap introduced into a template structure is assessed based on the number of lost contacts. The user can easily implement one’s own scoring function by extending one of the provided abstract classes [30].

## Results and discussion

Here we use the application in two real life examples to demonstrate the robustness and possible applications of the software. The scripts used in the experiments with the relevant input data were published on the project website.

### Threading as a structural alignment algorithm

**Figure 5:**
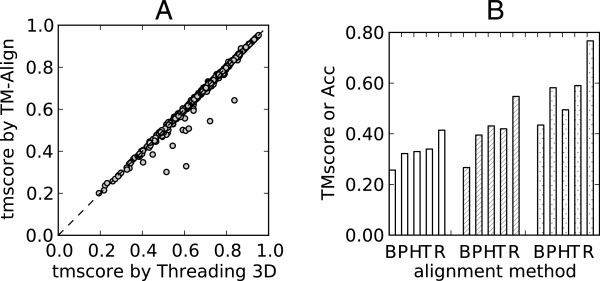
**Example results.(A)** REMC-Threading algorithm (abscissa) as a structure alignment software compared to TM-align (ordinate) on more than 1000 protein pairs. **(B)** Comparison between **B**LOSUM62, **P**ICASSO3, **H**HAlign, **T**hreading1D and **R**EMC-Threading: white bars - average TMscore, striped bars - average Al_0*P*_, dotted bars - average Al_4*P*_ alignment quality on MALIDUP set. The best value for all the three score types is 1.0.

Threading where both the query and the template protein structures are known is equivalent to the protein structural alignment. The calculation of such alignments is a perfect test for searching strategies. In the Bioshell-Threading package there are three scoring functions which can be used for this purpose: TM-score, RMSD and DALI-score. In this contribution we compare the TM-align [17] algorithm with 3D threading in which -TMscore [17] was used as an energy function. The benchmark set [38] comprises more than 1000 pairs of homologous proteins. REMC simulation was performed for 10 replicas with temperatures distributed from 0.08 to 10.0 dimensionless units. It can be be seen in the Figure 5A that alignments found by REMC search are in most cases very close to TM-align results. In a very few cases, however, 3D threading can find a significantly better match which suggests that the heuristic search implemented in TM-align, although very fast, does not always find the optimal solution. The calculations were repeated with the fragment-based variant of TM-align (fr-TM-align) [39] which resolved virtually all of the discrepancies. On the other hand, other structural alignment tools such as CE or DALI yielded alignments with worse TM-scores (data not shown). This was expected since these tools were designed to optimize their own Z-scores rather than the TM-score parameter. It should be noted that this parameter was arbitrarily chosen in this experiment to test sampling efficiency. Searching with BioShell-Threading also generates sub-optimal structural alignments which are often very close (within 0.01 TM-score), but may differ significantly from the optimal solution (data not shown).

### Quality of query sequence-template structure alignments

To test the Threading algorithm on more realistic problems, the MALIDUP [40] benchmark has been used. Results are shown in the Figure 5B. MALIDUP benchmark comprises 241 protein pairs of diverged duplicated domains. It was chosen because the evolutionary relation for the domains under consideration is fairly recognized and not biased by sequence similarity. The 3D-Threading algorithm was compared with four other methods: global sequence alignment with the BLOSUM62 matrix (optimized in [38]), profile-to-profile alignment (optimized in [38]) with the PICASSO3 [34] scoring function, Threading1D [38,41] and the widely used, state-of-the-art method: HHAlign [10,42,43]. For profile-to-profile, threading 1D and threading 3D runs sequence profiles were generated with five PsiBlast [44] iterations against the NR90 database and e-value threshold below 0.00001. For the HHalign algorithm, multiple sequence alignments (MSA), were created in the local searching mode with hhblits[43] on the NR20 database created on January 10, 2011. Subsequently, these MSAs were used in aligning query and template sequences with hhalign, in the global alignment mode.

The following scoring terms were used: EnvScore, ProbabilisticSecondaryScore, PICASSO3, GoLikeScore, TwoBodyContact with Miyazawa-Jernigan contact scoring and StrcGapPenalty. The following weights: 0.1, 0.25, 0.5, 1.0, 0.4 and 0.15, respectively were optimized on the ProSup [45] dataset. The objective of this test was the quality of calculated alignments *i.e.* average TM-score and alignment overlap with manually curated alignments. The latter was measured as the percent of correctly aligned positions AL_0_P and the fraction of aligned positions predicted with an error of at most four positions AL_4_P. For the MALIDUP set it can be observed that the threading algorithm, which incorporates 3D information from the template structure (column ‘**R**’ in the Figure 5B), performs better than the other tested algorithms, both in respect of average TM-score and overlap with manual alignments (as assessed by AL_0_P and AL_4_P scores). Profile-based aligners: Threading 1D and HHAlign perform comparably on these benchmarks, whereas BioShell-Threading performs much better. In particular, when compared to HHAlign (column ‘**H**’), it achieves approximately 0.09 higher AL_0_P and 0.24 higher AL_4_P. This is partially due to the fact, that in case of unrecognized homology, HHAlign returns a null alignment (which affects the averaged score value). There are some possible applications of this result. It can be used to generate alignment boundaries for protein modeling algorithms which can use such the information [32,46]. In this case the alignment boundary is the range for every query’s residue to which it can align within the template structure. It is also possible, using sub-optimal alignments, to create more diverse spatial constraints for algorithms such as Modeller [1].

### Practical considerations

The computational approach utilized in this contribution is an example of a stochastic simulation rather than a typical alignment method. User has to define a number of parameters to control this process, such as the number of Monte Carlo replicas and the respective set of their temperatures. Fortunately, several methods have been devised for REMC parameters selection, e.g. [32,47]. In general, these parameters depend both on query and template proteins and should be optimized separately for each case. However, for the sake of simplicity, for any benchmark calculation presented in this contribution we used the same set of ten replicas as described above. This temperature set is wide enough to obtain good results for all the test cases but inevitably increases the computational effort. Optimization of these parameters might also occasionally lead to better alignments. However, even for the optimal set of parameters it takes from several minutes to more than an hour to reliably sample the low energy area of the alignment space. The three-dimensional threading Monte Carlo simulation will always be at least an order of magnitude slower than a dynamic programming calculation but is usually faster than RAPTOR - another three dimensional threading where calulations even for short sequences take more than an hour [48]. RAPTOR method however employs branch-and-bound approach and, unlike a stochastic simulation, the reach of the global optimum of a scoring function is guaranteed. Other parameters a user should optimize are: scoring function weights and probabilities of particular alignment modifications (i.e. moves). The extensive study of this parameter space is beyond the scope of this paper and will be described elsewhere. In this work, to avoid overtraining, we optimized the scoring function and movers set on ProSup data set [49], which has not been used for benchmarking purposes.

The 3D threading application can also be used as a structural alignment method. In the presented benchmark, it has been compared with TMAlign and yielded nearly the same results. CPU time required by TMAlign was however about two orders of magnitude shorther (minutes to an hour by the threading vs seconds by TMAlign). This result is a direct consequence of the number of times each of the two programs calls the TM-score evaluation routine. In order to test the convergence of the threading, calculations were started from a random alignment. During the simulation TM-score has to be evaluated at every Monte Carlo move which, unlike scores derived from ByAtomEnergy, cannot be recalculated locally just for the moved block. TMAlign, on the other hand, starts from an alignment computed by a dynamic programming procedure and evaluates TM-score a few times until convergence is reached. The threading simulation however provides a numer of different suboptimal alignments which all fall within 0.01 range in TM-score units.

## Conclusions

BioShell-Threading implements a three-dimensional protein threading algorithm based on a Monte Carlo search scheme. The code has been written in Java language which makes it virtually machine independent. It implements numerous scoring (energy) functions. Some of them can be applied in regular Dynamic Programming. For others, the optimization becomes a NP-hard problem and demand more time consuming methods (e.g. MC). The package provides a ready to use command-line application and a Java software library. This makes BioShell-Threading a component that can be very easily incorporated into larger protein structure pipelines [50]. By providing suboptimal alignments, the package can increase the accuracy of widely used protein folding softwares and proteins structure classification methods. However, the main goal of BioShell-Threading is the refinement of query-to-template alignments. At the time, when fold recognition methods are fast and quite accurate, alignment accuracy is the limiting factor. Thus using certain fast algorithms [10,41,44] to search the whole protein databases and then refine top hits with more sophisticated scoring function seems to be of a great value to the protein modeling community.

## Availability and requirements

• **Project name:** Bioshell Threading 3D

• **Project home page:**http://www.bioshell.pl/threading3d

• **Operating system(s):** Platform independent

• **Programming language:** Java

• **Other requirements:** Java 1.4 or higher

• **License:** Creative Commons by-sc-nd

• **Any restrictions to use by non-academics:** licence required

## Competing interests

The authors declare that they have no competing interests.

## Authors’ contributions

PG, AK, AK, DG developed the idea and the framework of the software. PG and DG implemented the software and carried out analyses. All authors wrote, read and approved the final manuscript.

## References

[B1] Sali A Blundell TL Comparative protein modelling by satisfaction of spatial restraints J Mol Biol 1993 234 779 815 8254673 10.1006/jmbi.1993.1626

[B2] Kolinski A Protein modeling and structure prediction with a reduced representation Acta Biochimica Polonica 2004 51 349 371 15218533

[B3] Zhang Y I-TASSER server for protein 3D structure prediction BMC Bioinformatics 2008 9 40 18215316 10.1186/1471-2105-9-40 PMC2245901

[B4] Kallberg M Wang H Wang S Peng J Wang Z Lu H Xu J Template-based protein structure modeling using the RaptorX web server Nat Protocols 2012 7 1511 1522 10.1038/nprot.2012.085 PMC4730388 22814390

[B5] Lathrop RH The protein threading problem with sequence amino acid interaction preferences is NP-complete Protein Eng 1994 7 1059 68 7831276 10.1093/protein/7.9.1059

[B6] Gront D Kolinski A BioShell - a package of tools for structural biology computations Bioinformatics 2006 22 621 622 16407320 10.1093/bioinformatics/btk037

[B7] Gront D Kolinski A Utility library for structural bioinformatics Bioinformatics 2008 24 584 585 18227118 10.1093/bioinformatics/btm627

[B8] Marti-Renom MA Madhusudjan MS Sali A Alignment of protein sequences by their profiles Protein Sci 2004 13 1071 87 15044736 10.1110/ps.03379804 PMC2280052

[B9] Zhou H Zhou Y Fold recognition by combining sequence profiles derived from evolution and from depth-dependent structural alignment of fragments Proteins 2005 58 321 328 15523666 10.1002/prot.20308 PMC1408319

[B10] Soding J Protein homology detection by HMM–HMM comparison Bioinformatics 2005 21 951 960 15531603 10.1093/bioinformatics/bti125

[B11] Lobley A Sadowski MI Jones DT pGenTHREADER and pDomTHREADER: new methods for improved protein fold recognition and superfamily discrimination Bioinformatics 2009 25 1761 1767 19429599 10.1093/bioinformatics/btp302

[B12] Chen H Kihara D Effect of using suboptimal alignments in template-based protein structure prediction Proteins 2010 79 315 34 21058297 10.1002/prot.22885 PMC3058269

[B13] Mirny LA Shakhnovich EI Protein structure prediction by threading. why it works and why it does not? J Mol Biol 1953 283 507 526 9769221 10.1006/jmbi.1998.2092

[B14] Metropolis N Rosenbluth AW Rosenbluth MN Teller AH Teller E Equations of state calculations by fast computing machines J Chem Phys 1953 21 1087 1092

[B15] Kirkpatrick S Gelatt CD Vecchi MP Optimization by simulated annealing Science 1983 220 671 680 17813860 10.1126/science.220.4598.671

[B16] Swendsen RH Wang JS Nonuniversal critical dynamics in Monte Carlo simulations Phys Rev Lett 1987 58 86 88 10034599 10.1103/PhysRevLett.58.86

[B17] Zhang Y Skolnick J TM-align: a protein structure alignment algorithm based on the TM-score Nuc Acids Res 2005 33 2302 2309 10.1093/nar/gki524 PMC1084323 15849316

[B18] Miyazawa S Jernigan RL Estimation of effective interresidue contact energies from protein crystal structures: quasi-chemical approximation Macromolecules 1985 18 534 552

[B19] Miyazawa S Jernigan RL Residue-residue potentials with a favorable contact pair term and an unfavorable high packing density term, for simulation and threading J Mol Biol 1996 256 623 644 8604144 10.1006/jmbi.1996.0114

[B20] Holm L Sander C Protein structure comparison by alignment of distance matrices J Mol Biol 1993 233 123 38 8377180 10.1006/jmbi.1993.1489

[B21] Kabsch W A solution of the best rotation to relate two sets of vectors Acta Crystallogr 1976 32 922 923

[B22] Taketomi H Ueda Y Go N Studies on protein folding, unfolding and fluctuations by computer simulation Int J Pept Prot Res 1975 7 445 459 1201909

[B23] Tegge A Wang Z Eickholt J Cheng J NNcon: Improved protein contact map prediction using 2D-recursive neural networks Nucl Acids Res 2009 37 W515 W518 19420062 10.1093/nar/gkp305 PMC2703959

[B24] Godzik A Kolinski A Skolnick J Are proteins ideal mixtures of amino acids? analysis of energy parameter sets Protein Sci 1995 4 2107 17 8535247 10.1002/pro.5560041016 PMC2142984

[B25] Skolnick J Jaroszewski L Kolinski A Godzik A Derivation and testing of pair potentials for protein folding: when is the quasichemical approximation correct? Protein Sci 1997 6 676 688 9070450 10.1002/pro.5560060317 PMC2143667

[B26] Skolnick J Kolinski A Ortiz A Derivation of protein-specific pair potentials based on weak sequence fragment similarity Proteins 2000 38 3 16 10651034

[B27] Vendruscolo M Domany E Pairwise contact potentials are unsuitable for protein folding J Chem Phys 2004 109 11101 11108

[B28] Eyal E Frenkel-Morgenstern M Sobolev YV Pietrokovski S A pair-to-pair amino acids substitution matrix and its applications for protein structure prediction Proteins 2007 67 142 53 17243158 10.1002/prot.21223

[B29] Miyazawa S Jernigan RL Identifying sequence–structure pairs undetected by sequence alignments Protein Eng 2000 13 459 475 10906342 10.1093/protein/13.7.459

[B30] Bioshell’s Documentation website [http://www.bioshell.pl/~git/biosimulations.doc/html]

[B31] Chang I Cieplak M Dima R Maritan A Banavar JR Protein threading by learning Proc Natl Acad Sci 2001 98 14350 14355 11717394 10.1073/pnas.241133698 PMC64685

[B32] Gront D Kolinski A Efficient scheme for optimization of parallel tempering Monte Carlo method J Phys: Condens Matter 2007 19 3 036225 036234. [http://dx.doi.org/10.1088/0953-8984/19/3/036225]

[B33] Wang G Dunbrack RL Scoring profile-to-profile sequence alignments Protein Sci 2004 13 1612 1626 15152092 10.1110/ps.03601504 PMC2279992

[B34] Mittelman D Sadreyev R Grishin NV Probabilistic scoring measures for profile-profile comparison yield more accurate short seed alignments Bioinformatics 2003 19 1531 1539 12912834 10.1093/bioinformatics/btg185

[B35] Yona G Levitt M Within the twilight zone: a sensitive profile-profile comparison tool based on information theory J Mol Biol 2002 315 1257 7 11827492 10.1006/jmbi.2001.5293

[B36] Henikoff S Henikoff JG Amino acid substitution matrices from protein blocks Proc Nat Ac Sci 1992 89 10915 10919 10.1073/pnas.89.22.10915 PMC50453 1438297

[B37] Dayhoff MO Schwartz RM Chapter 22: A model of evolutionary change in proteins Atlas of Protein Sequence and Structure 1978

[B38] Gniewek P Kolinski A Gront D Optimization of profile-to-profile alignment parameters for one-dimensional threading J Comp Biol 2012 19 879 886 10.1089/cmb.2011.0307 22731622

[B39] Pandit SBB Skolnick J Fr-TM-align: a new protein structural alignment method based on fragment alignments and the TM-score BMC Bioinformatics 2008 9 531+ 19077267 10.1186/1471-2105-9-531 PMC2628391

[B40] Cheng H Bong-Hyun K Grishin NV MALIDUP: A database of manually constructed structure alignments for duplicated domain pairs Proteins 2008 70 1162 6 17932926 10.1002/prot.21783

[B41] Gront D Blaszczyk M Wojciechowski P Kolinski A Bioshell Threader: protein homology detection based on sequence profiles and secondary structure profiles Nucl Acids Res 2012 23 2522 2527 10.1093/nar/gks555 PMC3394251 22693216

[B42] Farrar M Smith-Waterman speeds database searches six times over other SIMD implementations Bioinformatics 2007 23 156 61 17110365 10.1093/bioinformatics/btl582

[B43] Remmert M Biegert A Hauser A Soding J HHblits: Lightning-fast iterative protein sequence searching by HMM-HMM alignment Nat Methods 2011 25 173 5 22198341 10.1038/nmeth.1818

[B44] Altschul SF Madden TL Schaffer AA Zhang J Zhang Z Miller W Lipman DJ Gapped BLAST and PSI-BLAST: a new generation of protein database search programs Nucleic Acids Res 1997 25 3389 3402 9254694 10.1093/nar/25.17.3389 PMC146917

[B45] Lackner P Koppensteiner WA Sippl MJ Domingues FS ProSup: a refined tool for protein structure alignment Protein Eng 2000 13 745 752 11161105 10.1093/protein/13.11.745

[B46] Trojanowski S Rutkowska A Kolinski A TRACER. A new approach to comparative modeling that combines threading with free-space conformational sampling Act Bioch Pol 2010 57 125 133 20309433

[B47] Trebst S Troyer M Hansmann UHEH Optimized parallel tempering simulations of proteins J Chem Phys 2006 124 17 174903 174908, [http://dx.doi.org/10.1063/1.2186639] 16689600 10.1063/1.2186639

[B48] Xu J Li M Kim D Xu Y RAPTOR: optimal protein threading by linear programming J Bioinform Comput Biol 2003 1 95 117. [http://view.ncbi.nlm.nih.gov/pubmed/15290783] 15290783 10.1142/s0219720003000186

[B49] Domingues FS Lackner P Andreeva A Sippl MJ Structure-based evaluation of sequence comparison and fold recognition alignment accuracy J Mol Biol 2000 297 4 1003 1013 10736233 10.1006/jmbi.2000.3615

[B50] Kmiecik S Jamroz M Zwolinska A Gniewek P Kolinski A Designing an automatic pipeline for protein structure prediction NIC Series 2008 40 105 108

